# Physical Properties of Normal Grade Biodiesel and Winter Grade Biodiesel

**DOI:** 10.3390/ijms12042100

**Published:** 2011-03-25

**Authors:** Amir Reza Sadrolhosseini, Mohd Maarof Moksin, Harrison Lau Lik Nang, Monir Norozi, W. Mahmood Mat Yunus, Azmi Zakaria

**Affiliations:** 1 Department of Physics, Faculty of Science, Universiti Putra Malaysia 43400 UPM Serdang, Selangor, Malaysia; E-Mails: maarof@science.upm.edu.my (M.M.M.); monir.noroozi@gmail.com (M.N.); mahmood@science.upm.edu.my (W.M.M.Y.); azmizak@science.upm.edu.my (A.Z.); 2 Malaysian Palm Oil Board, No. 6 Persiaran Institusi, Bandar Baru Bangi, 43000 Kajang, Selangor, Malaysia; E-Mail: harrison@mpob.gov.my

**Keywords:** normal biodiesel, winter biodiesel, surface plasmon resonance, Photopyroelectric (PPE) technique, palm oil biodiesel

## Abstract

In this study, optical and thermal properties of normal grade and winter grade palm oil biodiesel were investigated. Surface Plasmon Resonance and Photopyroelectric technique were used to evaluate the samples. The dispersion curve and thermal diffusivity were obtained. Consequently, the variation of refractive index, as a function of wavelength in normal grade biodiesel is faster than winter grade palm oil biodiesel, and the thermal diffusivity of winter grade biodiesel is higher than the thermal diffusivity of normal grade biodiesel. This is attributed to the higher palmitic acid *C*_16:0_ content in normal grade than in winter grade palm oil biodiesel.

## 1. Introduction

Nowadays, biodiesel is the best candidate to replace petroleum-based fuel. Biodiesel is an available alternative fuel for diesel engines. It is a renewable, non-toxic and low emission [1–3] fuel. Cloud point and pour point are two significant parameters used to evaluate the biodiesel fuel. The cloud point is the Wax Appearance Temperature (WAT) or Wax Precipitation Temperature (WPT) [4], the temperature at which dissolved solids appear in diesel or biodiesel fuel. The pour point is the lowest temperature at which the fuel can flow [4]. These parameters depend on the concentration of *C*_16:0_, *C*_18:0_ and *C*_18:1_ in the product. These are the index of the biodiesel quality and show the ability of the biodiesel for application at low temperatures. The palm oil contains 32–45% Palmitic acid (*C*_16:0_), 2–7% Stearic acid (*C*_18:0_), 38–52% Oleic acid (*C*_18:1_) and 5–11% Linoleic acid (*C*_18:2_). If in production of biodiesel, the new esters are derived from saturated fatty acids such as *C*_16:0_ and *C*_18:0_, the biodiesel has a high cloud point and pour point because they are a precursor for crystallization.

The response of material to light beams depends on real and imaginary parts of the refractive index, and the absorption coefficient is related to the imaginary part. Moreover, when the biodiesel temperature is near to the cloud point, a cloudy state appears, and the refractive index changes; hence, it is a significant parameter to evaluate the state of a biodiesel. In addition, refractive index also depends on the concentration of saturated and unsaturated fatty acids. Thus, it is a useful parameter for standardization of the product.

In this study, the winter grade palm oil biodiesel and normal grade palm oil biodiesel were characterized with Surface Plasmon Resonance (SPR) and Photopyroelectric (PPE) techniques. The refractive index and the thermal diffusivity of the biodiesels were measured and compared at room temperature.

## 2. Theory

### 2.1. Surface Plasmon Resonance (SPR)

The SPR is an optical phenomenon related to a charge density oscillation at the interface between two materials which have the real parts of their dielectric constant of opposite sign [5]. The interaction of light and surface plasmon can be investigated using Fresnel reflection theory. Now, we assume the metal layer is sandwiched between prism and dielectric layer (biodiesel sample) and the incident wave is in the *y*-*z* plan so the amplitude of reflected light *A_r_* can be expressed as:

$$A_{r}=r_{123}A_{i}$$

where *A_i_* is the amplitude of the incident light, and *r*_123_ is the amplitude reflection coefficient which depends on thickness (*t*) as follows:

(1) $$r_{123}=\frac{r_{12}+r_{23}\;\text{exp}(2{\text{ik}}_{2x}t)}{1+r_{12}\;\text{exp}(2{\text{ik}}_{2x}t)}$$

where *r*_12_, *r*_23_ and *k*_2*x*_ are the reflection coefficient of metal dielectric, the reflection coefficient of glass metal and the phase constant [6], respectively. The *k_ix_* and *k_jx_* are

(2) $$k_{\text{ix}}=\sqrt{{\left(\frac{2\pi }{\lambda }\right)}^{2}\varepsilon_{i}-k_{z}^{2}},\;\;\;k_{\text{jx}}=\sqrt{{\left(\frac{2\pi }{\lambda }\right)}^{2}\varepsilon_{j}-k_{z}^{2}}$$

Where the *k_z_* is the propagation constant ( $\frac{2\pi }{\lambda }n_{p}\;\text{sin}\theta_{2}$), and *r_ij_* is

(3) $$r_{\text{ij}}=\frac{\varepsilon_{j}k_{\text{ix}}-\varepsilon_{i}k_{\text{jx}}}{\varepsilon_{j}k_{\text{ix}}+\varepsilon_{i}k_{\text{jx}}},\;\;\;i,j=1,2,3$$

The reflectivity is *R* = |*r*_123_|^2^.

The condition of resonance depends on the refractive index of gold and the sample [6] as follows:

(4) $$n_{p}\;\text{sin}\theta_{R}=\sqrt{\left(n_{1}^{2}n_{2}^{2})/(n_{1}^{2}+n_{2}^{2}\right)}$$

where *θ_R_*, *n_p_*, *n*_1_ and *n*_2_ are the resonance angle, refractive indices of the prism, gold layer and sample, respectively. The refractive index of the sample is

(5) $$n_{2}=\sqrt{\left(n_{1}^{2}n_{p}^{2}\;{\text{sin}}^{2}\theta_{R})/(n_{1}^{2}-n_{p}^{2}\;{\text{sin}}^{2}\theta_{R}\right)}$$

If *A* is the angle of the prism and *θ*_1_ is the angle of incidence of the light beam directed to the prism, the angle of incidence on the metal layer is obtained as follows [7]:

(6) $$\theta_{2}=A-\text{arcsin}\left(\left(n_{\text{air}}/n_{p}\right)\text{sin}\theta_{1}\right)$$

where *n_air_* is the refractive index of air.

The reflectivity is a function of refractive index of sample, refractive index and thickness of gold layer. When the thickness and optical parameters of the gold layer are known, the angle of incidence at the interface between the prism and the gold layer is obtained from Equation (6). The refractive index and resonance angle of the sample will be found by minimizing the sum [8]

(7) $$\Gamma =\sum_{\theta }\left[R_{\text{Exp}}(\theta_{2},n_{2})-R_{\text{Theory}}(\theta_{2},n_{2})\right]$$

where *R_Exp_* and *R_Theory_* are the experimental and theoretical reflectivity, respectively. The reflectivity is a function of angle and wavelength.

### 2.2. Photopyroelectric (PPE) Technique

The photopyroelectric (PPE) technique was used to measure the thermal diffusivity of winter grade and normal grade palm oil biodiesel. In the low frequency (modulated radiation) regime, the thermal wave penetrates into the sample, and the sensor gives rise to a pyroelectric signal. The sample becomes thermally thick and the pyroelectric (PE) signal *V*(*f*) decreases exponentially with increasing modulation of the frequency. The decay rate (*V*) is determined by the thickness and thermal diffusivity of the sample.

(8) $$V(f)=V_{0}\;\text{exp}\left[-(1+i)\sqrt{\frac{\pi f}{\alpha }}L\right]$$

(9) $$Ln|V(f)|=\left[Ln|V_{0}|-\sqrt{\frac{\pi }{\alpha }}L\sqrt{f}\right]$$

where *V*(*f*) is the complex PE signal, *V* is its amplitude factor, and *f* is the modulation frequency. *L*, *α* are the thickness of sample and thermal diffusivity, respectively. The frequency scan of the PE signal provides the direct and absolute measurement of the thermal diffusivity of the sample [9].

## 3. Methodology

### 3.1. SPR Setup

**Figure 1.**
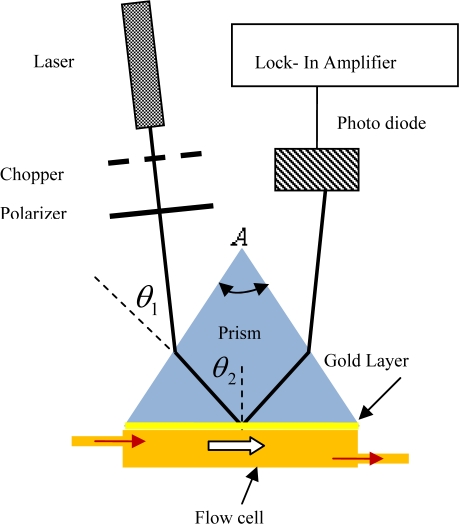
Experimental setup. (He-Ne laser, polarizer, prism, photodiode, flow cell, chopper and Lock in Amplifier).

The setup in Figure 1 consists of a precision rotation stage, a high refractive index prism (ZF 52, *n* = 1.83956, *A* = 60°, Foctek), a photodiode, a polarizer, a chopper, a lock-in amplifier, a He-Ne laser (632.8 nm, model: R30990, Newport) and a flow cell. The rotation stage and the photodiode were controlled with a program that was written with Matlab. In this setup, the rotation stage was connected to a stepper motor. At first, the prism was adjusted to its starting point before being rotated up to 40° with increments of 0.016° step size [10]. At each step, the rotation stage momentarily stopped for the reflected light intensity off the gold layer to be registered by the photodiode, which was connected to the lock-in amplifier. In addition, the angle of incidence between the air and the prism (*θ*_1_) is also registered.

All measurements were carried out with the biodiesel in direct contact with the gold layer. The thickness of the gold layer, which was deposited on the prism with sputtering coating, was 49 nm. In order to determine the optical parameters of the biodiesel samples, the SPR setup was calibrated using deionized water; hence, the optical parameters (*n*, *k*) of the gold layer were also obtained. The SPR experiment was repeated with He-Ne laser (594.1 nm Uniphase, model: 1675), He-Ne laser (543.5 nm, Uniphase, model: 1677) and diode laser (405 nm, Power Technology, model LDCU87870).

### 3.2. Photopyroelectric (PPE) Technique Setup

**Figure 2.**
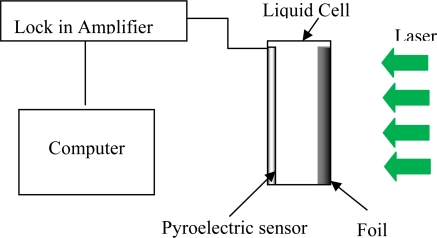
Schematic diagram of the photopyroelectric (PPE) technique setup. The thickness of sample, metal thin foil and sensor is 0.21 mm, 50 μm and 52 μm, respectively.

The PPE setup in Figure 2 consists of a pyroelectric sensor (Polyvinylidene Diflouride PVDF (MSI DT1-028K/L)), a diode laser (532 nm, 200 mw), a lock in amplifier, metal thin foil (50 μm) and a fluid cell. The laser beam impinges on the black-painted external face of thin metal foil. This face of metal foil absorbed the energy and converted it into heat. Thus, the thermal wave generated by the laser beam was transferred into the liquid. The pyroelectric sensor (52 μm) detected the signal which is very sensitive to small changes in the heat flux [11]. It was fixed to a perspex substrate, PE transducer and backing is in a thermally thick condition. Thus, the resulting thermal wave is independent of the other cell parameters. The electromagnetic noise was reduced by eliminating all the ground loops via proper grounding. The operating parameters are controlled through a computer equipped with adapted virtual instrument software that allows automatic data acquisition. The frequency range for the scan was 5 Hz to 40 Hz. The thermal diffusivity measurement was performed at room temperature (∼20 °C). A careful calibration of the experimental setup and procedure was done and verified by measuring the thermal diffusivity of water (standard), prior to carrying out the actual measurements.

### 3.3. Gas Chromatography (GC)

The winter grade biodiesel and normal grade biodiesel were analyzed by gas chromatography (GC). In this experiment, the fatty acid composition of methyl esters was determined using PerkinElmer GC-FID (Auto system XL, Shelton, CT, USA). The GC oven was kept at 140 °C and the helium gas was flowed in the fused silica column. Sodium methylate (0.3 mL) and hexane (1.0 mL) were added to 0.03 g of sample. The mixture was injected into GC with split ratio of 1:100.

### 3.4. Fourier Transform Spectroscopy (FTIR)

The samples were tested with Fourier Transform Infrared Spectroscopy (FTIR) model Elmer 1725X. The spectrum from 1000 to 4000 cm^−1^ was registered.

### 3.5. Sample Preparation

#### 3.5.1. Normal Grade Palm Oil Biodiesel (NPB)

Five hundred grams of refined palm oil was transesterified with 200 g of methanol and 0.6 wt% of sodium hydroxide. The reaction was carried out at 70 °C for one hour. The glycerol phase was drained as the bottom layer from the esters phase. Several portions of 250 mL of hot water were added to the ester layer until a neutralized phase was obtained. The esters layer was then nitrogen-pump dried under vacuum to obtain the final normal grade biodiesel.

#### 3.5.2. Winter Grade Palm Oil Biodiesel (WPB)

One hundred grams of normal palm oil methyl esters obtained from the above experiment were mixed with equal amount of methanol and chilled at 5 °C for 24 hours. The mixture was then filtered immediately with vacuum suction. Methanol was removed from the filtrate by nitrogen pump drying to obtain the final winter grade biodiesel.

The physical properties of the samples such as density, viscosity, cloud point, pour point, cetane number and acid value were measured according to ASTM D6751 and are listed in Table 1.

## 4. Results and Discussion

**Figure 3.**
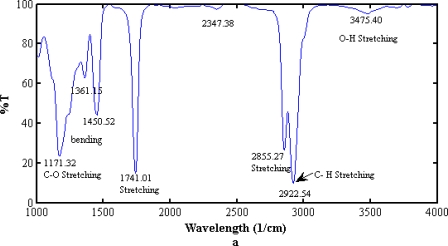

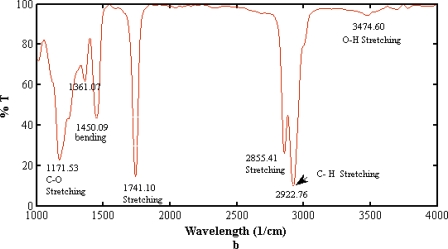
The Fourier Transform Infrared Spectroscopy (FTIR) spectra of (**a**) NPB and (**b**) WPB.

**Figure 4.**
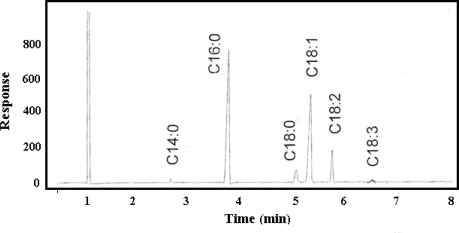
Chromatogram of normal grade palm oil biodiesel.

**Figure 5.**
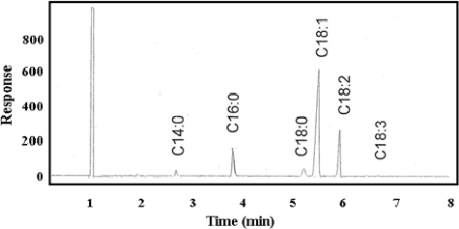
Chromatogram of winter grade palm oil biodiesel.

Figures 3a and 3b show the similar spectrum of FTIR for NPB and WPB biodiesel, respectively. Stretching and bending were found in both samples, which depend on methyl ester. Both spectra reveal the existence of functional group in the samples. The difference between the two biodiesels can be found in Figures 4 and 5, which show the chromatograms of NPB and WPB. Thus, the higher concentration of methyl ester of saturated fatty acid (*C*_16:0_) in NPB than in WPB results in the higher cloud point and pour point of NPB than those of WPB.

**Figure 6.**
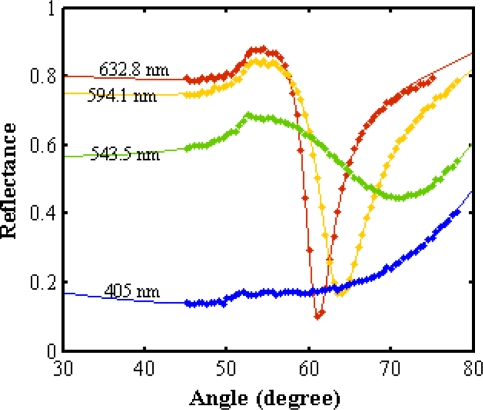
The Surface Plasmon Resonance (SPR) signal of deionized water to calibrate the SPR setup. The refractive index of the gold layer was 0.237+ 3.39*i*, 0.268 + 3.027*i*, 0.436 + 2.348*i* and 1.7293 + 1.8545*i* for 632.8 nm, 594.1 nm, 543.5 nm and 405 nm, respectively.

Figure 6 shows the SPR signals of deionized water which was utilized to determine the thickness and optical parameters of the gold layer. The refractive indices of deionized water are 1.3317, 1.333, 1.3344, and 1.3427 for the wavelengths 632.8 nm, 594.1 nm, 543.5 nm and 405 nm, respectively [12]. Thus, according to Equations (1) and (7) the optical parameters of the gold layer were achieved.

**Figure 7.**
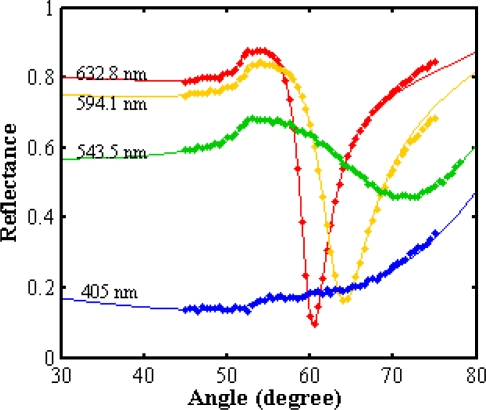
The SPR signal for normal grade palm oil biodiesel. ▪ and—are experimental data and fitted theoretical curve, respectively. The resonance angle and refractive index were obtained from fitting the theory to the experimental data.

**Figure 8.**
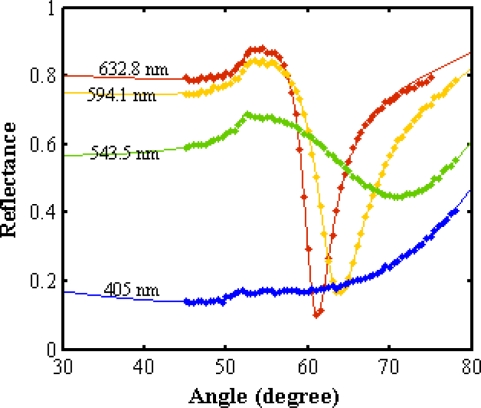
The SPR signal for winter grade palm oil biodiesel. ▪ and—are experimental data and fitted theoretical curve, respectively. The resonance angle and the refractive index were obtained from fitting the theory to experimental data.

**Figure 9.**
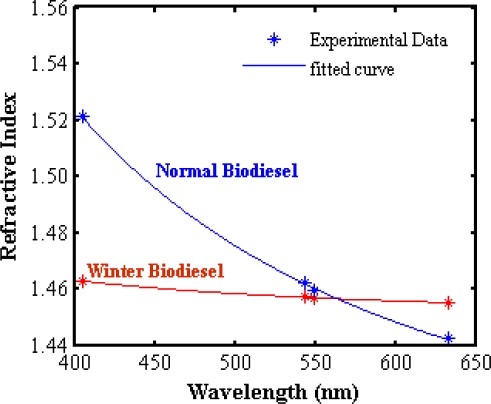
Dispersion curve of normal and winter biodiesel and the coefficients of Augustin Louis Cauchy [13] of NPB and WPB are as follows: NPB: b_1_ = −180.1, b_2_ = 181, c_1_ = −168 and c_2_ = 192.7; WPB: b_1_ = 4.234, b_2_ = −3.109, c_1_ =11.85 and c_2_ = 4.172.

Figures 7 and 8 show the SPR signals belonging to normal and winter grade palm oil biodiesel. The ranges of resonance angle of NPB and WPB are 60.39°–71.337° and 61.119°–70.945°, respectively. Consequently, the real (*n*) and imaginary parts (*k*) of the refractive indices are different. The pertinent parameters are summarized in Table 2. Figure 9 shows the dispersion curve of NPB and WPB. The slope of the dispersion curve of NPB is larger than the slope of dispersion curve of WPB. The results show that the variation of the refractive index of NPB with wavelength is faster than the variation of the refractive index of WPB. Since the measurements were carried out with monochromatic light and the light beam did not pass through the sample; the results were free from the effects of absorption of the light in the biodiesel sample and dispersion of the prism.

**Figure 10.**
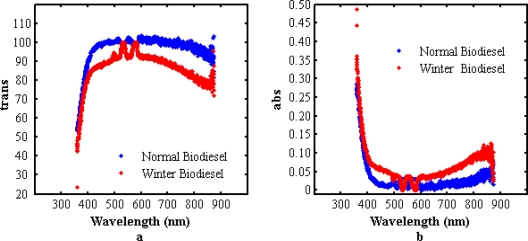
UV-Visible results of normal and winter grade palm oil biodiesel. (**a**) transmission; (**b**) absorption.

Figures 10a and 10b show the UV-Visible results of normal and winter grade palm oil biodiesel. The results show that the transparency of the NPB is higher than that of WPB, and as expected, the absorption of WPB is higher than NPB in the visible range.

**Figure 11.**
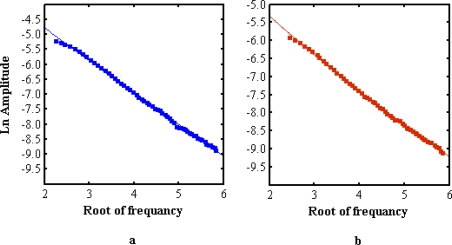
Experimental data and fitted frequency dependence of the amplitude of pyroelectric (PE) signal. (**a**) normal grade palm oil biodiesel (**b**) winter grade palm oil biodiesel.

Figure 11 shows a captured pyroelectric signal, plotted as the natural logarithm of the amplitude component *versus* the square root of the frequency. The slope of curves ( $V=\sqrt{\frac{\pi }{\alpha }}L$) depends on the thermal diffusivity and thickness. The thermal diffusivity values were calculated from the slopes of the linear part of the logarithmic amplitude of the signal curves. The pertinent parameters are sorted in Table 3. The results show that the thermal diffusivity of winter biodiesel and normal biodiesel are higher than the thermal diffusivity of palm oil 0.920 (× 10^−3^ cm^2^/s) [11].

Thermal diffusivity is the ratio of thermal conductivity to volumetric heat capacity. The thermal diffusivity data is therefore a measure of how quickly the biodiesel can respond to variations of temperature. Material of high thermal diffusivity has the ability to conduct heat faster than it can absorb and hence is a great help in distributing heat uniformly throughout the bulk of the material. Even though WPB has higher thermal diffusivity as compare to NPB, its value is still lower than the thermal diffusivity of water. The data therefore does not predict WPB may have a lower pour point than NPB.

The significant difference between NPB and WPB is shown in their dispersion curves. As refractive index relates to the dielectric permittivity of materials, NPB is considered highly dispersive medium as compare to WPB; hence, it is more vulnerable to the generation of dipole oscillations under the influence of an electric field. This may be due to the much higher *C*_16:0_ content in NPB than in WPB.

## 5. Conclusions

We have successfully characterized the winter and normal grade palm oil biodiesel both optically and thermally for the first time by using SPR and photopyroelectric (PPE) techniques. These are noninvasive, nondestructive and environmentally friendly methods for determining the thermal and optical characteristics of the biodiesel fuels. Consequently, the type of ester affected the optical and thermal properties of biodiesel. Hence, these methods are suitable techniques to recognize the type of biodiesel fuel.

## Figures and Tables

**Table 1.** The properties of normal grade palm oil biodiesel (NPB) and winter grade palm oil biodiesel (WPB).

**WPB**		**NPB**
Viscosity at 40 °C	4.423	4.415
Density at 15 °C	870.0	878.3
Cloud Point	−18.0	15.2
Pour Point	−21	15
Cetane Number	53.0	58.3
Acid Value	<0.5	<0.5

**Table 2.** The pertinent parameters of Surface Plasmon Resonance (SPR) signals for normal and winter grade palm oil biodiesel.

	**NPB**			**WPB**		

	***n***	***k***	*θ_R_*	***n***	***k***	*θ_R_*
**632.8 nm**	1.44643	0.00002	60.39°	1.45513	0.00003	61.119°
**594.1 nm**	1.45963	0.00004	64.143°	1.45683	0.000024	63.866°
**543.5 nm**	1.46225	0.00006	71.337°	1.45708	0.000023	70.945°
**405 nm**	1.52113	0.0003		1.46283	0.00036	

**Table 3.** Pertinent parameters of the photopyroelectric measurement. The standard error of thermal diffusivity (Δ*α*) was calculated from $\Delta \alpha =2\alpha (\frac{\Delta V}{V}+\frac{\Delta L}{L})$; where Δ*L* = 0.001 mm, Δ*V* = 0.005.

**Sample**	**Slope**	**Thermal diffusivity** (cm^2^/s) × 10^−3^	**Error Δα** (cm^2^/s) × 10^−3^
**Water**	0.977	1.448	0.01
**Winter Grade Biodiesel**	1.015	1.342	0.02
**Normal Grade Biodiesel**	1.114	1.114	0.01
